# Journey through Crohn’s Disease Complication: From Fistula Formation to Future Therapies

**DOI:** 10.3390/jcm10235548

**Published:** 2021-11-26

**Authors:** Federica Rubbino, Luana Greco, Alessio di Cristofaro, Federica Gaiani, Stefania Vetrano, Luigi Laghi, Stefanos Bonovas, Daniele Piovani

**Affiliations:** 1 Laboratory of Molecular Gastroenterology, IRCCS Humanitas Research Hospital, Via Manzoni 56, 20089 Rozzano, Italy; luana.greco@humanitasresearch.it (L.G.); alessio.dicristofaro@humanitasresearch.it (A.d.C.); luigiandreagiuseppe.laghi@unipr.it (L.L.); 2 Department of Medicine and Surgery, University of Parma, Via Gramsci 14, 43126 Parma, Italy; federica.gaiani@unipr.it; 3 Gastroenterology and Endoscopy Unit, University-Hospital of Parma, Via Gramsci 14, 43126 Parma, Italy; 4 Department of Biomedical Sciences, Humanitas University, Via Rita Levi Montalcini 4, 20072 Pieve Emanuele, Italy; stefania.vetrano@hunimed.eu (S.V.); daniele.piovani@humanitasresearch.it (D.P.); 5 IRCCS Humanitas Research Hospital, Via Manzoni 56, 20089 Rozzano, Italy

**Keywords:** Crohn-associated fistula, mucinous adenocarcinoma, epithelial-to-mesenchymal transition

## Abstract

Crohn’s Disease (CD) is a chronic inflammatory disorder in which up to 50% of patients develop fistula within 20 years after the initial diagnosis, and half of these patients suffer perianal fistulizing disease. The etiopathogenesis of CD-related perianal fistula is still unclear, and its phenotypical and molecular characteristics are even more indefinite. A better understanding would be crucial to develop targeted and more effective therapeutic strategies. At present, the most accredited theory for the formation of CD-related fistula identifies the epithelial-to-mesenchymal transition (EMT) as the driving force. It has been well recognized that CD carries an increased risk of malignancy, particularly mucinous adenocarcinoma is often associated with long-standing fistula in CD patients. Despite the availability of multiple treatment options, perianal fistulizing CD represents a therapeutic challenge and is associated with an important impact on patients’ quality of life. To date, the most effective management is multidisciplinary with the cooperation of gastroenterologists, surgeons, radiologists, and nutritionists and the best recommended treatment is a combination of medical and surgical approaches.

## 1. Common Features of Fistulae in Crohn’s Disease

**Figure 1:**
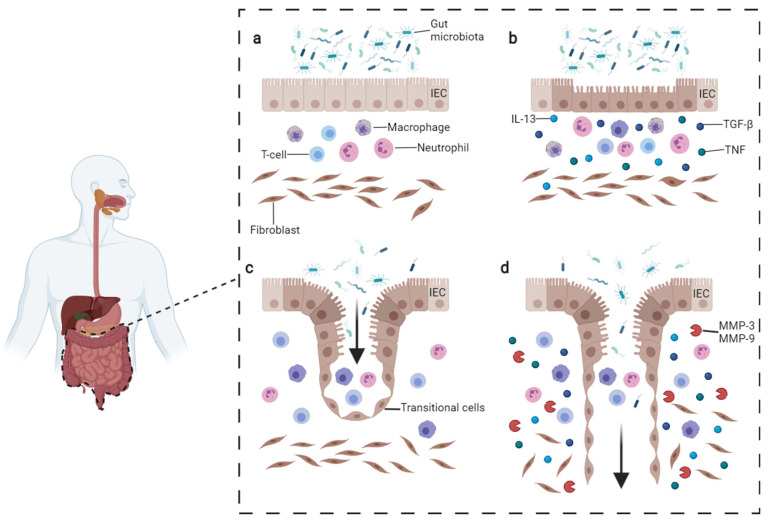
Pathogenesis of CD fistula. (**a**) Normal condition persisting in the gut. (**b**) In CD, the intestinal lesions can be triggered by multiple factors such as inflammatory cells reacting to microbiota or intestinal pathogens. The inflammatory infiltrate includes T-cells, B-cells, and macrophages which produce TNFα. Fibroblasts, staying underneath, produce TGF-β and IL-13. These cytokines trigger EMT and tissue remodeling. (**c**) During EMT, intestinal epithelial cells (IECs) lose their adhesion properties, downregulating β-catenin and E-cadherin proteins. IECs start migrating underneath in order to repair the lesion and become transitional cells (TCs). (**d**) TCs produce IL-13 which induces other cells to undergo EMT, penetrating deeper in lower layers. The process is facilitated by the production of metalloproteinases (MMPs) which degrade the extracellular matrix. Image adapted from Panes et al. [15] and drawn with BioRender.com (accessed on 23 November 2021).

Crohn’s Disease (CD) is a chronic inflammation in which the disorder could affect any region of the gastrointestinal tract from the mouth to the anus (but with typically higher incidence on ileum and colon), and together with Ulcerative Colitis (UC) are stand for inflammatory bowel diseases (IBD). Although the precise beginning remains unknown, the starting point of CD is believed to result from the interplay between genetic susceptibility, environmental factors, and their interactions with gut microbiota [1,2]. Moreover, the interaction between these factors and the patient’s immune system is as well considered crucial for the pathogenesis of the disease (Figure 1a,b).

The clinical presentation of CD depends substantially on the location and severity of the inflammation. Symptoms and signs can be heterogeneous, ranging from anorexia, abdominal pain, and fatigue, to more severe conditions as rectal bleeding, bloody diarrhea, and development of perianal lesions such as ulcerations and fistulas [3]. About 50% of CD patients are reported to develop perianal fistula in no more than 20 years after the initial diagnosis, and half of these patients suffer perianal fistulizing disease [2]. Although CD treatment is constantly improved by new therapeutic tools, the optimization of existent therapies and the adoption of a multidisciplinary approach to fistula management remains a clinical challenge [4,5]. This is mainly due to a minority of patients achieving remission and sometimes due to the lack of availability of a few specific therapies and feasibility of surgical techniques [6]. 

CD-related fistula can be distinguished by the presence of a central fissure that invaginates deeply toward the mucosal barrier of the gut, surrounded by neutrophils, lymphocytes, and granulation tissue. The fence of the fistula canal is densely populated by T-cells CD45RO positive, macrophages CD68 positive, and B-cell CD20 positive. 

Morphologically, the fistula is a tract between two surfaces lined by epithelial cells and loaded with debris, erythrocytes, and since it is a consequence of chronic inflammation—inflammatory infiltrates and fibrotic cells [7]. A reduced epithelial regeneration [7], together with weak migration capability of colonic lamina propria fibroblasts (CLPF) are responsible for the wound healing failure in CD-fistulizing/penetrating phenotype [8]. 

In order to restore the integrity of the intestinal epithelial barrier, the defect in CLFP migration is compensated by intestinal epithelial cells (IECs) converting into transitional cells (TCs), which gain mesenchymal myofibroblast-like features through a mechanism called epithelial-to-mesenchymal transition (EMT) [9] (Figure 1c,d). In this complex program, IECs lose cell polarity and adherence while acquiring invasive and migratory properties (see below).

Another important factor possibly contributing to the pathogenesis of CD-related fistula is the gut microbiota. The hypothesis is indirectly supported by the fact that antibiotic therapy might be beneficial for treating fistulizing CD. A study carried out during GEMINI II clinical trial revealed that patients treated with Vedolizumab and antibiotic therapy had a three-times higher chance of fistula closure than those on placebo [10]. Moreover, Fecal Microbiota Transplantation (FMT) seems to have a role in maintaining Crohn’s disease remission [11]. Haac and colleagues [12] highlighted a difference in the gut microbiota of patients with CD-fistula phenotype in comparison to other CD phenotypes. An abundance of opportunistic pathogens like *Achromobacter, Corynebacterium*, *Actinomyces,* and *Fusobacterium* was found in samples obtained from CD patients. Noticeably, *Fusobacterium nucleatum* was recently associated with colorectal cancer subtypes in several studies [13,14]. This evidence underlines the significant role that microorganisms, either pathogens or commensals, could play in the onset and/or maintenance of perianal fistulizing CD. 

## 2. Signature Differences Distinguish CD-Related Fistulae from Idiopathic Ones

Although there are several recognized risk factors for Crohn’s disease [1], no environmental factors have been associated with the etiopathogenesis of CD-related fistula yet. In fact, CD-related fistula appears mostly related to genetic, microbiological, and immunological components [16]. Furthermore, its phenotypical and molecular characteristics are not well-characterized.

Few comparative studies have allowed to identify markers able to distinguish CD fistulae from non-inflammatory bowel disease ones (also known as idiopathic or crypto-glandular). 

The crypto-glandular type was first classified, albeit inconclusively, by Parks in 1961 [17] on the basis of histological evidence of infection in anal glands. 

For a long time, it was thought that the persistence of the fistula depended upon inflammation, the presence of bacterial endotoxins perpetuating an inflammatory response within the lumen even when bacteria are destroyed [18], and upon the presence of bacterial pro-inflammatory peptidoglycan (PG) which may stimulate the secretion of interleukin-1β (IL-1β) [19]. 

The first evaluations on specific inflammatory characteristics of the fistulae were carried out by immunohistochemistry (IHC). CD fistulae diverge considerably from non-CD fistulae concerning their inflammatory composition, except for the lining epithelium. CD fistulae present high levels of infiltrated T cells CD45RO positive in the interior wall, dense infiltration of B cells CD20 positive surrounding lined up by a small group of macrophages CD68 positive. The second type has an intense infiltration of CD68 positive macrophages enclosed by CD45RO positive T lymphocytes [8]. 

Metalloproteases (MMP) and tissue inhibitors of metalloproteinases (TIMPs), which contribute to fistula formation through extracellular matrix degradation, did not show any difference between the two types of fistulae, with exception of MMP-3 and 9 that appear to be upregulated in idiopathic fistula [20]. 

Abundant expression of IL-1β, IL-8, IL-12p40 cytokines, and tumor necrosis factor (TNF)-α were found in idiopathic anal fistulae [21]. However, TNF-α was also significantly up-regulated in the serum of CD-related perianal fistula patients [22].

Only a subgroup of both fistula types showed a layer with easily recognizable epithelial cells which have tight junctions and a basal membrane. Of interest, the “non-epithelialized” CD fistulae were covered by a fine layer of myofibroblast-like “transitional cells” (TC) with gap junctions [7]. Their epithelial origin was strongly suggested by the cytokeratins (CK) 8 and 20 positivity, as well as vimentin negativity. In the transitional zone between the epithelium and the TC, β6-Integrin, and TGF-β had the highest staining intensities [10], as well as the expression of succinate receptor (SUCNR1) which seems to be correlated with the expression of Integrin Subunit Beta 6 (ITGB6), SNAIL, SLUG, and Vimentin [23]. These evidences suggested and supported again the involvement of EMT process in fistula formation [9].

Data on inflammation and EMT were confirmed also in non-CD fistulae through real-time PCR (RT-PCR) and Western blot analysis beside IHC. The inflammation was evaluated by the expression of IL-8 and IL-1β that were respectively more expressed in the proximal part than in the distal one and vice versa for the second marker [24,25]. The expression of markers such as TGF-β, Vimentin, ZEB1, SNAIL which were highly expressed in both proximal and distal parts, with mild E-cadherin reduction, were used for EMT evaluation [25].

Antimicrobial peptides, that are controlled in response to a bacterial antigens provocation or the presence of inflammatory cytokines, specifically hBD2 and hBD3 together with RNase7 and psoriasin, resulted to be elevated in the distal area of non-CD fistulae [24].

Furthermore, flow cytometry analysis (FACS) on curettage and tissue biopsy of CD-related fistulae do not present differences in the ratio of CD161^+^, IL-17^+^IFN-γ^−^, IL-17^−^IFN-γ^+^ and IL-17^+^IFN-γ^+^ cells between CD4^+^ or CD8^+^ T cells. CD161^+^ T lymphocytes were instead more expressed [26].

Proteomic analysis was performed to evaluate potential dissimilarity in cytokines and phosphoprotein concentration. The phosphorylation status of 28 Receptor Tyrosine Kinases (RTKs) and 11 signaling nodes in addition to 30 cytokines and chemokines was quantified. The two types of fistulae do not substantially differ in their protein expression pattern, even though the panel of cytokines and phosphoproteins analyzed was huge [27].

Recently, by metabolomic analysis, CD perianal fistula tissue has shown metabolic variations compared to idiopathic fistula tissue. Using two analytical platforms such as ultra-high-performance liquid chromatography system and a mass spectrometry detector coupled with chromatography, a broad coverage of the metabolome was achieved. Investigated markers occurred in pathways involved in many metabolisms such as amino acids, carnitine, phospholipids, lysine degradation, sphingolipids, glycerophospholipids, and purines. In CD fistula, dimethylarginine and decanoyl-l-carnitine were identified as the most significant predictors, whereas decanoy-l-carnitine was found to be decreased and positively correlated with idiopathic fistula. Lipid profiling revealed hexosylceramide and diglyceride, which belong to glycosphingolipids and diacylglycerol lipid classes, were found to be increased in CD fistulae with respect to idiopathic ones. A different class of compounds lipid-like called acylcarnitines, is implicated in lipid and fatty acid metabolism as well as cell signaling, playing a role in maintaining membrane integrity, and possibly in fistula persistence. Some of these acylcarnitines (e.g., carnitine precursor deoxycarnitine, lysine) are also thought to be determinant in EMT [28], but how this could be linked to specific metabolic changes remains unclear. It is plausible that metabolic changes accompanying EMT precede some of the more obvious signals of mesenchymal transition and indeed actively contribute to the activation of these indicators [29].

**Figure 2:**
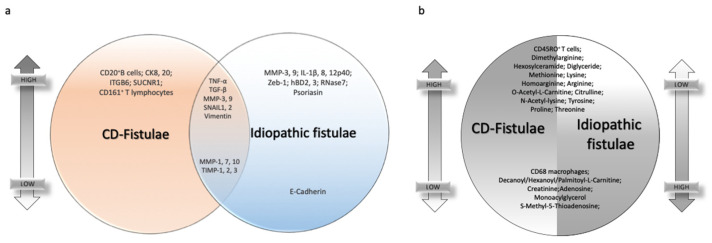
Common and dissimilar signatures between CD- and idiopathic fistulae. (**a**) Venn diagram highlighting individual or common markers expression in CD-related fistulae and idiopathic ones, on the top if they are upregulated, on the bottom if they are downregulated. (**b**) Differential markers expression in CD-related fistulae versus idiopathic fistulae. On the right of the circle, the expression is lower on the top of the graph and higher on the bottom. Vice versa, on the left part of the circle, the expression of the same markers is higher on the top and lower on the bottom.

All described signatures are schematized in Figure 2.

## 3. EMT Process in Fistula Formation

Even though the clinical relevance of perianal fistulizing CD is obvious due to its impact on patients’ life conditions, its etiopathogenesis is poorly understood. A better understanding would be crucial to develop targeted and more effective therapeutic strategies. 

The most accredited theory for the formation of CD-related fistula identifies the epithelial-to-mesenchymal transition (EMT) as the driving force. A large number of EMT-associated events can be noted within and around the fistula tract. During EMT, epithelial cells lose their epithelial-specific features, such as apico-basal polarity and cell contacts, acquiring a mesenchymal cell shape, as well as enhanced motility and dissemination [30]. Consistently, epithelial markers (such as E-cadherin and Claudin-4) were found downregulated, while mesenchymal markers (such as alpha smooth muscle actin and vimentin) [10] resulted upregulated. Further evidences demonstrated a strong expression of SNAIL in the nuclei of TCs, but not in the fistula surrounding area, supporting the increased activity of EMT-related Transcription Factors (TFs) [31]. Around the CD-related fistula, also one other TF, such as SLUG, was found to be increased, but its distribution was different from that of SNAIL. SNAIL was detectable only in TCs, whilst SLUG was more pronounced in cells surrounding the fistula tract [31]. 

In a separate study, the same group demonstrated that TGF-β not only induces *SNAI1* expression but also *IL-13* at mRNA level in the fibroblasts of colonic lamina propria derived from fistulizing CD patients [32]. This expression was even validated at protein level in TCs covering the fistula tract, and in intestinal epithelial cells (IEC) of misshapen adjacent crypts. At the same time, IL-13 was scarcely detectable in specimens from active UC and basically absent in non-IBD mucosa controls. This observation was unexpected because, normally, IL-13 is expressed by immune cells [33], and correlates with fibrosis [34,35,36]. At the functional level, it has been proven that IL-13 causes an increased expression of genes associated with cell invasion into TCs [32], suggesting a synergistic step-by-step process whereby TGF-β induces EMT by causing epithelial disruption, possibly as part of regular wound healing during chronic intestinal inflammation, and IL-13 finally facilitate the EMT cells to penetrate into deeper tissue layers. 

Along this migratory potential, the expression of Dickkpof-Homolog-1 (Dkk-1) [37,38] was also checked in fistulizing CD patients [39]. While Dkk-1 had weak expression in non-IBD controls, its expression was higher in the TCs layer and fibrotic area surrounding fistula, as well as in patients with active IBD [39].

Another study shows the increase of EMT phenomenon in patients with penetrating CD via an increased interaction between the Frizzled Class Receptor 4 (FDZ4) and the Wnt Family Member 2B (WNT2B). WNT2B induced the mRNA expression of c*MYC, VIM, SNAI1*, and *SNAI2*, but decreasing *CDH1* one. In addition, WNT2B induced the phosphorylation of STAT3 in cells derived from intestinal crypts [40]. In patients with stricturing CD, the greater Wnt activation through cytoskeleton rearrangement and cell movement [10], might be also involved in the gain of EMT detection.

Ortiz-Masia and colleagues [23] demonstrated that in penetrating CD patients the SUCNR1 expression and succinate levels are increased in the fistula tract. More specifically, succinate is involved in the variation of Wnt ligands expression, activating the signaling cascade, and consequently provokes EMT.

In perianal fistulae, the accumulation of CD4^+^ CD161^+^ T-cells with a Th17, Th1, and both phenotypes have been described [23]. Other studies showed an altered balance between MMPs and their tissue inhibitors (TIMPs), resulting in aberrant extracellular matrix degradation [20]. Specifically, MMP-3 and 9 (expressed mostly in mononuclear cells and granulocytes respectively) were upregulated in the CD fistula area, while TIMP-1 to 3 expression was lower compared to normal colon tissue [21]. Differently, other types of MMPs such as MMP-1, MMP-2, MMP-7, and MMP-10 are downregulated or not expressed [20]. It is also important to mention that immunotherapy against MMP-9 seems to prevent intestinal fibrosis among CD complications [41].

Finally, in the last published work, patients with fistulizing CD showed more CD3^+^ CD8^−^- and this subset produced higher amounts of IL-13- and less CD3^+^ CD8^+^ T cells in blood compared to healthy donors. Particularly, around the fistula tract, CD4^+^ cells were highly present. Then, both cell subsets promoted the expression of EMT-related genes when co-cultured with HT-29 cells [42].

However, the knowledge of fistula pathogenesis and the precise contribution of the EMT process remain nowadays limited, and further studies are needed to better understand the process of fistula development.

## 4. Mucinous Adenocarcinoma as a Complication in CD Patients with Fistula

The risk of malignancy in CD patients is well recognized. Particularly, three types of cancers arise more frequently in patients with CD than in the general population: small bowel cancer, colorectal cancer, and mucinous carcinoma arising from perianal fistulae [43]. The mucinous adenocarcinoma (MA) represents approximately 2–3% of large bowel cancers [44], and even though is a rare disease, it is often associated with long-standing CD-fistula [45,46], with an increased incidence in the last 20 years [46]. Up to date, only a small number of MA arising from a chronic anorectal fistula have been described in the literature, especially in patients without CD, in case reports or small series [47]. The lack of a sufficient number of patients for trials makes it difficult to conduct functional studies, as well as to progress in diagnosis and treatment, surgical resection remaining the first choice.

To confirm the association between chronic perianal fistula and MA, Santos et al. [48] published a case report showing that a patient with persistent perianal fistulae developed as a para-rectal tumor. Then, the presence of MA was confirmed through histopathological biopsy. 

Hugen et al. [49] listed the different causes of mucinous adenocarcinoma, such as genetic alterations, lifestyle variations, dietary changes, IBD, and radiotherapy, without including chronic anal fistula as a leading cause. 

In 2018 [50], a group from India tried to explain the origin of MA from chronic anal fistula. In this study, ultrasound-guided biopsy and histopathology proved the development of MA in a patient with chronic fistula. Particularly, they highlighted that the persistence of the fistula from 4 to 10 years was consistently associated with the diagnosis.

Similarly, Diaz-Vico [51] reported three patients in whom chronic perianal fistula tissue gave rise to the development of a mucinous adenocarcinoma over time. The gold standard for the diagnosis remains histopathology, and it is characterized by the presence of extracellular mucinous lakes encompassed by well-differentiated dilated tortuous glands, nerves, and vessels.

In 2015, a case report aimed to clarify the root of the mucinous adenocarcinoma associated with chronic perianal fistula in CD patients [52]. In this study, the mucosal biopsies collected from the lesions showed granulomatous tissue infiltrated by mucus-producing adenocarcinoma. The study supported the hypothesis that the constant mucosal regeneration inside CD-related fistula is a common pathogenetic mechanism for developing carcinoma. At the same time, immunosuppressants and anti-TNF agents may also facilitate the malignant transformation.

The research in this field has various limitations that could affect the accuracy of the results. Foremost, the number of affected patients is scarce, thus making it impossible to get sufficient data. Most investigations were based on small samples. Another important limitation is that the condition is difficult to diagnose until the onset of symptoms, and the very long latency makes the researcher bored or interrupt the study. 

## 5. Therapeutic Approaches to CD-Related Fistulae 

Perianal fistulizing CD is a particularly challenging form of CD, presenting in up to one-third of the patients [53]; in a tiny percentage of cases, it can be the only manifestation of the disease and may precede by several years intestinal manifestations of CD in up to 10% of the patients [54]. The presence of fistulae is often associated with an aggressive form of CD, with chronic course and disappointing rates of long-lasting remission [55].

As a consequence, patients commonly experience a negative impact on quality of life, including intimate and social relationships and a frequent need for hospital admissions and medical observations [6].

For these reasons, the scientific community is constantly searching for new therapeutic tools, with the aim to respond to this unmet clinical need.

Current guidelines recommend multidisciplinary management with the cooperation of gastroenterologists, surgeons, radiologists, and nutritionists, and suggest a combined medical and surgical approach as the most effective treatment for complex perianal fistulae in CD [56,57].

The establishment of biologic therapy has dramatically improved the efficacy of medical treatment of CD fistulae compared to the previous use of traditional immunomodulators, and, at present, anti-TNFα represent the therapy of choice in these patients [56,58]. The effectiveness of biologic therapy basically depends on the capability of these drugs to reduce tissue inflammation, which is the driving mechanism for fistulae development.

Medical therapy alone has demonstrated remission rates around 60%, and its combination with surgery improves reaction, recurrence rate, as well time to recurrence [59]. 

Anyway, immunosuppression by anti-TNF agents needs to evaluate the presence of abscesses (and the possibility to resolve them by drainage and antibiotics), due to their potential septic complications. 

The use of anti-TNFα has also been studied as to its local/topic injections, with the aim to potentiate the efficacy on fistula healing [60,61,62]. Although several reports have shown promising results, this technique has not been standardized yet, therefore it can be intended as a supportive tool in case other approaches have failed or are not available.

The use of biologics other than anti-TNFα (ustekinumab and vedolizumab) is not currently recommended as first-line therapy and should be considered only in case of contraindications to anti-TNFα [56].

Surgical treatment of perianal fistula associated with CD has the aim to adjuvate medical treatment, favoring fistula healing without compromising fecal continence. Depending on the type and extension of the fistula, the surgical approach can vary from simple drainage to more complex techniques [57]. The most common intervention is the application of setons to drain collections and control pelvic sepsis. This procedure should always be combined with medical therapy [54,63].

The closure of the fistula tract can be attempted by using different techniques, either endoscopic or surgical, and materials including fibrine glue, plugs, and n-butyl-2-cyanoacrylate (Histoacryl). Among these, fibrin glue injection is the most common technique, with a good safety profile and limited costs, although penalized by limited efficacy [64]. The insertion of fistula plugs has also been tested; the procedure consists of the application of a bio-absorbable xenograft which should promote tissue regeneration and fistula closure. This technique has demonstrated a success rate equal to seton drainage [65], hence it is not recommended. 

Considering endoscopic techniques, fistula closure can be achieved by clipping with either through-the-scope or over-the-scope clips [66]. Clipping is effective in fistula closure, being a safe and simple procedure in the hands of trained endoscopists.

The advancement flap is probably the most used among the surgical techniques. The procedure was first developed for treating cryptoglandular fistulae but is now routinely applied also in CD patients. Fistula healing is complete in about half of the patients [67].

Of comparable efficacy, ligation of the intersphincteric fistula tract (LIFT), which was also initially described in the treatment of cryptoglandular fistulae and has been then transferred to CD associated fistulae [54]. Consisting of the opening of the intersphincteric groove, dissection, and isolation of the fistulous tract, ligation of the tract with interrupted sutures and closure of the perianal wound LIFT is a demanding procedure and should always be performed by dedicated surgeons. Compared to advanced flap procedure, LIFT has demonstrated lower incontinence rates (7.8% versus 1.6%) [68].

In order to preserve the sphincter functionality, the video-assisted anal fistula treatment (VAAFT) consists of the video-assisted inspection of the fistula followed by a precise cauterization of the fistula tract from the external towards the internal margin, and closure of the internal opening. Although not yet routinely applied in CD fistula surgery, this technique appears promising, especially for the benefits of sphincteric preservation [69]. Similar to VAAFT, the fistula laser closure technique applies laser instead of electrocautery and is not performed under direct vision. This technique has similar efficacy to VAAFT but shorter learning curves [70].

Overall, surgery for CD fistulae should always be performed by expert operators in high-flow centers after adequate study of the clinical case. Local availability and expertise should guide the choice of the technique.

The limited success rate of combined medical and surgical therapy, although being slightly improved, has promoted the research of novel methods. One of the most promising is the injection in the fistula tract of mesenchymal stem cells (MSCs), aimed at tissue regeneration using a minimally invasive procedure [71]. 

MSCs are non-hematopoietic multipotent cells, which can be set apart from connective tissues, like adipose tissue, and from the bone marrow. These cells have been studied in fistula treatment due to their immunomodulatory, immunosuppressive, and regenerative properties [72]. Their use as treatment of CD fistulae has been described by Panés et al. in the ADMIRE trial [73] with promising results in terms of efficacy and safety, which paved the way to their entrance also in the last ECCO guidelines [57].

The progressive in-depth analysis on the pathogenetic mechanisms of CD fistulae has allowed hypothesizing new promising therapeutic tools, such as anti-MMP antibodies. Studies about anti-MMP drugs start from the assumption that MMP-9, a type IV collagenase, has a central role in tissue remodeling and is upregulated in crypt abscesses and around fistulae [74]. A study by Fontani et al. [75] showed in vitro that N-acetylcysteine and curcumin were able to downregulate MMP-3 in high oxidative state conditions, and specifically in TNFα stimulated cells, suggesting that such antioxidants may have a therapeutic use for the prevention and treatment of fistulae in the gut of CD patients.

Another study by Goffin et al. [41] was conducted in vitro from human specimens and in mice xenograft, confirming in the patients affected by penetrating CD the upregulation of MMP-9, and showing in mice a protective effect of anti-MMP antibodies with respect to intestinal fibrosis. Albeit in literature only animal- and in vitro studies are available, the future application of such molecules could revolutionize the treatment of perianal fistulae.

The understanding of the crucial role of inflammation in fistula development has sustained the still limited but promising application of hyperbaric oxygen therapy as supportive treatment in patients affected by perianal CD [76]. The treatment consists of breathing 100% oxygen under increased atmospheric pressure, provoking tissue hyperoxygenation and oxidative stress which has been associated to stem cell mobilization and upregulation of growth factors and ultimately to anti-inflammatory effects. Considering its safety and limited costs if the equipment is available, this treatment appears a valid supportive method for patients with otherwise unsatisfactory healing [77].

## 6. Conclusions

Despite the availability of multiple treatment options, perianal fistulizing CD represents a therapeutic challenge and is associated with an important impact on patients’ quality of life. To date, the most effective management is multidisciplinary with the cooperation of gastroenterologists, surgeons, radiologists, and nutritionists and the best recommended treatment is a combination of medical and surgical approaches. In the last decades, the broadened knowledge about fistulae pathogenesis has allowed a surge of novel therapeutic tools: some are already applied, such as MSCs injection, some others show potentiality, such as the administration of anti-MMP antibodies. Overall, the best therapeutic option should be always tailored to the patient’s clinical condition. Based on the variable response to standard treatments, a new supportive tool should be considered.

## Data Availability

Not applicable.
